# Correction: Impact of early-onset persistent stunting on cognitive development at 5 years of age: Results from a multi-country cohort study

**DOI:** 10.1371/journal.pone.0229663

**Published:** 2020-02-20

**Authors:** Md Ashraful Alam, Stephanie A Richard, Shah Mohammad Fahim, Mustafa Mahfuz, Baitun Nahar, Subhasish Das, Binod Shrestha, Beena Koshy, Estomih Mduma, Jessica C Seidman, Laura E Murray-Kolb, Laura E Caulfield, Aldo A. M Lima, Pascal Bessong, Tahmeed Ahmed

Aldo A. M. Lima and Pascal Bessong are not included in the author byline. Aldo A. M. Lima should be listed as the thirteenth author and affiliated with Clinical Research Unit and Institute of Biomedicine, Federal University of Ceara, Fortaleza, Brazil. The contributions of this author are as follows: Conceptualization, Data Curation, Investigation, Methodology and Writing – Review & Editing. Pascal Bessong should be listed at the fourteenth author and affiliated with the University of Venda, Thohoyandou, South Africa. The contributions of this author are as follows: Conceptualization, Data Curation, Investigation, Methodology and Writing – Review & Editing.
