# Supplementary material for: Impact of early-onset persistent stunting on cognitive development at 5 years of age: Results from a multi-country cohort study
Source: PLoS One. 2020 Jan 24;15(1):e0227839. doi: 10.1371/journal.pone.0227839 (PMC6980491; doi:10.1371/journal.pone.0227839)
Supplement: S1 Table — (DOCX) [file pone.0227839.s002.docx]

**S1 Table. Institutional Research Boards Approvals.**

| **Nepal** | |
| --- | --- |
|  | Institute of Medicine, TU; Institutional Review Board |
|  | Nepal Health Research Council; Ethical Review Board |
|  | Walter Reed Army Institute of Research; Institutional Review Board |
| **South Africa** | |
|  | University of Venda; Health, Safety and Research Ethics Committee |
|  | Limpopo Provincial Government; Dept of Health and Social Development |
|  | University of Virginia; Institutional Review Board for Health Sciences Research |
| **Tanzania** | |
|  | National Institute for Medical Research; Medical Research Coordinating Committee |
|  | Ministry of Health and Social Welfare; Chief Medical Officer |
|  | University of Virginia; Institutional Review Board for Health Sciences Research |
| **India** | |
|  | Christian Medical College; Institutional Review Board |
|  | Indian Council of Medical Research; Health Ministry Screening Committee |
| **Brazil** | |
|  | Universidade Federal do Ceara; Committee for Ethics in Research |
|  | Health Ministry, Council of National Health; National Ethical Research Committee |
|  | University of Virginia; Institutional Review Board for Health Sciences Research |
| **Bangladesh** | |
|  | ICDDR,B; Ethical Review Committee |
|  | University of Virginia; Institutional Review Board for Health Sciences Research |
