# Supplementary material for: Impact of early-onset persistent stunting on cognitive development at 5 years of age: Results from a multi-country cohort study
Source: PLoS One. 2020 Jan 24;15(1):e0227839. doi: 10.1371/journal.pone.0227839 (PMC6980491; doi:10.1371/journal.pone.0227839)
Supplement: S2 Table — (DOCX) [file pone.0227839.s003.docx]

S2 Table. Comparison of children retained in the cohort through five years of age (‘Include’) and those lost-to-follow up or with incomplete data (‘Exclude’).

| Variables | BGD | | BRF | | INV | | NEB | | SAV | | TZH | |
| --- | --- | --- | --- | --- | --- | --- | --- | --- | --- | --- | --- | --- |
|  | Include (n=186) | Exclude (n=79) | Include (n=105) | Exclude (n=128) | Include (n=211) | Exclude (n=40) | Include (n=125) | Exclude (n=115) | Include (n=176) | Exclude (n=138) | Include (n=140) | Exclude (n=122) |
| ^a^ Male children, n (%) | 95 (51.08) | 34 (43.04) | 56 (53.33) | 64  (50) | 98 (46.45) | 15 (37.50) | 57 (45.60) | 73* (63.48) | 89 (50.57) | 66 (47.83) | 74 (52.86) | 55 (45.08) |
| ^b^ Birth weight in kg,  mean ± SD | 2.82  ± 0.41 | 2.73  ± 0.41 | 3.33  ± 0.50 | 3.35  ± 0.47 | 2.89  ± 0.44 | 2.84  ± 0.39 | 3.00  ± 0.41 | 2.95  ± 0.38 | 3.15  ± 0.46 | 3.10  ± 0.47 | 3.30  ± 0.44 | 3.15*  ± 0.47 |
| ^c^ Maternal education (years), median (IQR) | 5  (2,7) | 5  (1,7) | 9  (8,12) | 9  (7,12) | 8  (4,9) | 9*  (7,10) | 10 (5,12) | 9  (6,10) | 11  (9,12) | 11  (9,12) | 7  (3,7) | 7  (3,7) |
| ^c^ Asset score, median (IQR) | 3 (2,5) | 3 (2,4) | 7 (7,8) | 7 (6,7) | 3 (2,5) | 4 (2,5) | 6 (5,7) | 6 (4,7)* | 7 (5,8) | 7 (5,8) | 2 (1,3) | 1 (0,3) |
| ^c^ Income in USD, median (IQR) | 105  (74,138) | 98  (72,142) | 357  (314,408) | 327  (303,395) | 61 (44,100) | 66 (55,110) | 130 (94,207) | 139 (109,213) | 191  (119,299) | 185  (116,306) | 18  (9,31) | 12  (7,28) |
| ^a^ Improved drinking water,  n (%) | 186 (100) | 56  (100) | 105  (100) | 105  (100) | 211 (100) | 24  (100) | 125 (100) | 110  (99) | 153 (86.93) | 66 (81.48) | 43 (30.71) | 37 (33.64) |
| ^a^ Improved sanitation, n (%) | 186 (100) | 56  (100) | 103  (98) | 104  (99) | 101 (47.87) | 9 (37.50) | 125 (100) | 110  (99) | 171 (97.16) | 79 (79.53) | 9  (6.43) | 31* (28.18) |
| ^a^ ALRI incidence rate (times per child-year) | 0.48 | 0.47 | 0.1 | 0.1 | 1.56 | 1.23 | 0.85 | 1.01 | 0.3 | 0.29 | 0.3 | 0.29 |
| ^a^ Diarrheal incidence rate (times per child-year) | 3.65 | 3.39 | 0.54 | 0.4* | 2.09 | 2.24 | 2.37 | 2.25 | 0.64 | 0.63 | 1.23 | 1.45* |

^a^ Chi-squared test performed

^b^ T-test performed

^c^ Mann-Whitney test performed

* p-value<0.05
