# Supplementary material for: Impact of early-onset persistent stunting on cognitive development at 5 years of age: Results from a multi-country cohort study
Source: PLoS One. 2020 Jan 24;15(1):e0227839. doi: 10.1371/journal.pone.0227839 (PMC6980491; doi:10.1371/journal.pone.0227839)
Supplement: Attachment — Submitted filename: Response to Reviewers.docx [file pone.0227839.s004.docx]

Journal requirements:

1. When submitting your revision, we need you to address these additional requirements.

Please ensure that your manuscript meets PLOS ONE's style requirements, including those for file naming. The PLOS ONE style templates can be found at

[http://www.journals.plos.org/plosone/s/file?id=wjVg/PLOSOne_formatting_sample_main_body.pdf](https://nam01.safelinks.protection.outlook.com/?url=http%3A%2F%2Fwww.journals.plos.org%2Fplosone%2Fs%2Ffile%3Fid%3DwjVg%2FPLOSOne_formatting_sample_main_body.pdf&data=02%7C01%7Clem118%40psu.edu%7Cc4667a97aa5648cc0b1008d76848179d%7C7cf48d453ddb4389a9c1c115526eb52e%7C0%7C1%7C637092531192162635&sdata=tYnRcwc3TGHfJMghDw9KE7xmzHGtmdiBOoEjNclMVFQ%3D&reserved=0) and [http://www.journals.plos.org/plosone/s/file?id=ba62/PLOSOne_formatting_sample_title_authors_affiliations.pdf](https://nam01.safelinks.protection.outlook.com/?url=http%3A%2F%2Fwww.journals.plos.org%2Fplosone%2Fs%2Ffile%3Fid%3Dba62%2FPLOSOne_formatting_sample_title_authors_affiliations.pdf&data=02%7C01%7Clem118%40psu.edu%7Cc4667a97aa5648cc0b1008d76848179d%7C7cf48d453ddb4389a9c1c115526eb52e%7C0%7C1%7C637092531192172633&sdata=N0YIZEKwjEDmyeinEJxAkOTS34%2Fu1rWlrRZo1bXyZSY%3D&reserved=0)

***Response:*** *Thank you for the comment. We followed the style of PLOS ONE in the revised version.*

2. Thank you for including your ethics statement:

"Ethics approval was obtained from the institutional review boards at participating institutions (Supplement Table 1). Written informed consent was obtained from the parent or guardian of each participating child.".

i) Please amend your current ethics statement to include the full name of the ethics committee/institutional review board(s) that approved your specific study.

ii) Once you have amended this/these statement(s) in the Methods section of the manuscript, please add the same text to the “Ethics Statement” field of the submission form (via “Edit Submission”).

For additional information about PLOS ONE ethical requirements for human subjects research, please refer to [http://journals.plos.org/plosone/s/submission-guidelines#loc-human-subjects-research](https://nam01.safelinks.protection.outlook.com/?url=http%3A%2F%2Fjournals.plos.org%2Fplosone%2Fs%2Fsubmission-guidelines%23loc-human-subjects-research&data=02%7C01%7Clem118%40psu.edu%7Cc4667a97aa5648cc0b1008d76848179d%7C7cf48d453ddb4389a9c1c115526eb52e%7C0%7C1%7C637092531192172633&sdata=JEXUj9E71HGG5okjD6MDHlxZYUzID9G3VOEYaROIESY%3D&reserved=0).

***Response:*** *Thank you for the feedback. We have included the “Ethics Statement” in the methodology section.*

3.  Thank you for stating in your Funding Statement:

"This study was supported by the Bill and Melinda Gates Foundation. The funders had
no role in study design, data collection and analysis, decision to publish, or preparation
of the manuscript.".

i) Please provide an amended statement that declares *all* the funding or sources of support (whether external or internal to your organization) received during this study, as detailed online in our guide for authors at [http://journals.plos.org/plosone/s/submit-now](https://nam01.safelinks.protection.outlook.com/?url=http%3A%2F%2Fjournals.plos.org%2Fplosone%2Fs%2Fsubmit-now&data=02%7C01%7Clem118%40psu.edu%7Cc4667a97aa5648cc0b1008d76848179d%7C7cf48d453ddb4389a9c1c115526eb52e%7C0%7C1%7C637092531192182629&sdata=%2BcuPmMJZecOHlrofTW6EQrokuv6yvEos9kMA8SXQ0uk%3D&reserved=0).  Please also include the statement “There was no additional external funding received for this study.” in your updated Funding Statement.

ii) Please include your amended Funding Statement within your cover letter. We will change the online submission form on your behalf.

***Response:*** *Thank you for the feedback. We have amended the “Funding Statement” and included that in the manuscript and cover letter.*

Additional Editor Comments (if provided):
Abstract: Please state in bracket that the HOME inventory is an index of quality of the home environment. In the existing form it may confuse readers.

***Response:*** *This has been amended as per the suggestion.*

Background 87-88: “However, the meta-analysis was based on observational studies……”????? I don’t think it would be possible to determine the relationship between linear growth and cognitive development through experimental design. It is also important that the current study also followed observational design.

***Response:*** *Thank you for the feedback. We have revised this sentence.*

Methods: 106-7: was there any statistical basis for planning 200 children per site? Also, I see nothing about the sampling approach used for enrolling the children at baseline.

***Response:*** *Thank you very much for this very important question. The sample size calculation was described in one of the initial papers that presented the two-year findings:*

*“Enrollment took place over a 2-year period with the goal of enrolling 200 children per site. While this sample size was established as the maximum number of children who could be followed intensively at each site, we tested the power of the study a priori under varying rates of enteropathogen detection, proportions of incident malnutrition outcomes at 24 months, and effect sizes. Under the assumption that 8% of children would be malnourished at birth, the effective sample size was estimated at 184 children per site, or 1,472 children for all sites. Therefore, if 40% of the children became malnourished (i.e., a weight-for-age* Z*-score < −2 or height-for-age* Z*-score < −2) at the end of 2 years and if an enteropathogen infection affected 50% of the children over 2 years, we would have 60% power for a single site and 100% power for all sites combined to detect a 50% higher risk of being malnourished with 95% confidence. The power of the study to detect a 70% increase in malnutrition risk associated with an infection (all other assumptions being the same) was 82% for a single site and 100% for all sites combined. Additional details are provided elsewhere (*[*S2 Text*](https://www.ncbi.nlm.nih.gov/pmc/articles/PMC5656304/#pmed.1002408.s014)*) [1].*

*In addition, the sampling approaches are described in the original site-specific papers contained in an early MAL-ED study supplement [2-9]. We have also added some brief mention of these processes to the text.*

Line 145-151: please add a sentence or two that describes the sub-scales of the “HOME inventory”.

***Response:*** *Thank you so much for your comment. We have described the sub-scales of the “HOME inventory” in the methods section.*

I fear AGP measurement alone (I,e, without CRP) may not fully adjust the micronutrient markers for inflammation. It is not clear why the authors did not collect data of CRP too.

***Response:*** *While measurement of CRP was initially proposed, funding precluded us from measuring it. We agree that adjustment for micronutrient biomarkers would have been more complete, had we been able to measure CRP. We concur this is a limitation of the study and we have included this limitation in the manuscript.*

Discussion: Can you please discuss the possibility of over-adjustment bias in the model because impaired immunity and increased susceptibility to infection can be a pathway that links stunting with psychomotor development.

***Response:*** *Thank you for the feedback. We added these sentences to the limitation section: “In addition, some of the factors included in the model may be associated with stunting status and including them as covariates may lead to over-adjustment. More complex models may be planned in the future to account for interrelationships among the different risk factors.”*

Reviewers' comments:

Reviewer's Responses to Questions

**Comments to the Author**

1. Is the manuscript technically sound, and do the data support the conclusions?

The manuscript must describe a technically sound piece of scientific research with data that supports the conclusions. Experiments must have been conducted rigorously, with appropriate controls, replication, and sample sizes. The conclusions must be drawn appropriately based on the data presented.

Reviewer #1: Partly

Reviewer #2: Yes

Reviewer #3: Yes

2. Has the statistical analysis been performed appropriately and rigorously?

Reviewer #1: I Don't Know

Reviewer #2: Yes

Reviewer #3: Yes

3. Have the authors made all data underlying the findings in their manuscript fully available?

The [PLOS Data policy](https://nam01.safelinks.protection.outlook.com/?url=http%3A%2F%2Fwww.plosone.org%2Fstatic%2Fpolicies.action%23sharing&data=02%7C01%7Clem118%40psu.edu%7Cc4667a97aa5648cc0b1008d76848179d%7C7cf48d453ddb4389a9c1c115526eb52e%7C0%7C1%7C637092531192192619&sdata=RnWSsL2gWiuJOyKp5JIJYY1K1J4gAHCfP6qu2uk%2FvHk%3D&reserved=0) requires authors to make all data underlying the findings described in their manuscript fully available without restriction, with rare exception (please refer to the Data Availability Statement in the manuscript PDF file). The data should be provided as part of the manuscript or its supporting information, or deposited to a public repository. For example, in addition to summary statistics, the data points behind means, medians and variance measures should be available. If there are restrictions on publicly sharing data—e.g. participant privacy or use of data from a third party—those must be specified.
Reviewer #1: No

Reviewer #2: No

Reviewer #3: Yes

4. Is the manuscript presented in an intelligible fashion and written in standard English?

PLOS ONE does not copyedit accepted manuscripts, so the language in submitted articles must be clear, correct, and unambiguous. Any typographical or grammatical errors should be corrected at revision, so please note any specific errors here.

Reviewer #1: Yes

Reviewer #2: Yes

Reviewer #3: Yes

5. Review Comments to the Author

Please use the space provided to explain your answers to the questions above. You may also include additional comments for the author, including concerns about dual publication, research ethics, or publication ethics. (Please upload your review as an attachment if it exceeds 20,000 characters)

Reviewer #1: Plos one PONE_D_19_28331

This study uses data from 6 sites of the MAL-ED study to look at the association between stunting and cognitive scores at age 5 years.

The original study was very detailed, collecting extensive data on nutrition and illnesses as well as home background. In this context, the authors need to justify why it is useful to look at stunting – which is a result of those factors, and what their analysis adds to the papers already published using the same data

***Response:*** *Thank you very much for this very important question. The objective of the current study is to explore the association between early-onset persistent stunting in children and cognitive development at 5 years of age.*

*You have rightfully mentioned that extensive data on nutrition, illness and home background were collected in MAL-ED study and some of these variables were the predictors of childhood stunting [1].*

*Several papers have been published from MAL-ED network using birth to 60 months data. McCormick et al. 2019 examined the relation between early-life factors from birth to 2 years with cognitive development at 5 years of age. The objective of this paper was aligned with our current manuscript. However, this paper did not consider stunting status as one the predictors, nor did it consider the timing of stunting. Similar to this analysis, we also observed that plasma sTFR, home environment and socio-economic status were associated with cognitive status at 60 months of age. Childhood stunting has often been linked with poor cognitive outcomes in children, but the timing of the development of stunting, controlling for many other factors, is the interesting question this work explores. In this study we have further categorized stunting to identify particularly vulnerable groups, and this may help policy makers to efficiently advocate for additional resources.*

Furthermore, thought needs to be given to the likely causal pathways. Does it make sense to adjust for factors like the pathogen load, when this may be one of the causes of stunting? Adjusting for the causes of stunting risks over-adjustment.

***Response:*** *Thank you for the feedback. We added these sentences to the limitation section: “In addition, some of the factors included in the model may be associated with stunting status and including them as covariates may lead to over-adjustment. More complex models may be planned in the future to account for interrelationships among the different risk factors.”*

The outcome is the WPPSI score. I understand that this has been published previously but it would still be helpful to provide more information in this paper. How were the scales adapted for cultural appropriateness? Was this different in each country? How was it validated?

***Response:*** *Thank you for the feedback. We have added details to the methods section.*

I think – though it is not entirely clear from text or tables - that they used a measure of “fluid reasoning” rather than the whole WPSSI score. And that a constant scale was used across sites although the mean score was very different across the sites. Given these large differences, wouldn’t a relative within site scale be more appropriate for looking at the associations with stunting ?

***Response:*** *That is correct about the fluid reasoning and we have edited the text for clarity. The WPPSI was evaluated and a T-score was developed to compare children across sites. The background and further details about the factor structure and invariance across sites can be found in an article published in 2019 [10].*

And given that the proportion stunted varies so much by site, wouldn’t it be better to include site as a confounder than as a cluster variable?

***Response:*** *Thank you for the excellent suggestion - We ran the model both ways and the findings were almost the same. As we are not interested in differences by site except to control for them, we think that incorporating them as random effects is a better choice. Please see the model results in the below table:*

| **Site as a covariate:** | **Site as a random effect:** |
| --- | --- |
| Estimate Std. Error t value Pr(>\|t\|)  (Intercept) 41.57 6.33 6.57 0.00  Early-onset  persistent stunted -2.04 0.90 -2.28 0.02  Early-onset  only stunted -1.12 0.88 -1.28 0.20  Late-onset  persistent stunted -0.66 0.98 -0.67 0.50  Late-onset  only stunted -1.02 0.82 -1.24 0.22  sex 0.08 0.56 0.15 0.88  Birth weight 0.73 0.67 1.09 0.28  EBF -0.04 0.18 -0.20 0.84  ALRI 0.31 0.31 1.02 0.31  Diarrhea 0.03 0.17 0.16 0.87  Transferrin  receptor -0.33 0.09 -3.50 0.00  α-1 antitrypsin 0.91 0.74 1.23 0.22  Myeloperoxidase -0.21 0.62 -0.33 0.74  Bacteria load -0.07 0.10 -0.64 0.52  Virus load -0.27 0.38 -0.70 0.49  HOME score 0.45 0.13 3.51 0.00  WAMI index 1.49 0.25 5.97 0.00 | Value Std.Error t-value p-value  (Intercept) 41.90 6.70 6.25 0.00  Early-onset  persistent stunted -2.10 0.89 -2.35 0.02  Early-onset  only stunted -1.21 0.88 -1.37 0.17  Late-onset  persistent stunted -0.68 0.98 -0.70 0.49  Late-onset  only stunted -1.09 0.82 -1.32 0.19  sex 0.09 0.56 0.16 0.88  Birth weight 0.70 0.67 1.04 0.30  EBF -0.02 0.18 -0.10 0.92  ALRI 0.34 0.31 1.09 0.28  Diarrhea 0.04 0.17 0.22 0.82  Transferrin  receptor -0.31 0.09 -3.36 0.00  α-1 antitrypsin 1.02 0.74 1.38 0.17  Myeloperoxidase -0.27 0.61 -0.44 0.66  Bacteria load -0.06 0.10 -0.53 0.59  Virus load -0.28 0.38 -0.73 0.47  HOME score 0.46 0.13 3.64 0.00  WAMI index 1.50 0.24 6.18 0.00 |

There is no mention of possible interactions (by site or by other variables).

***Response****: Thank you so much for comment. We have tested for interaction and there was no evidence of interaction in terms of site and other variables. We have added this sentence in our manuscript’s Statistical analyses section.*

Some of the other variables also need more explaining:
What is the rationale for grouping water and sanitation, assets, maternal education and income into a composite score? Water and sanitation could have direct effects on infections and health which is different from the indirect effects of income and assets.

***Response:*** *Thank you so much for your comments. The WAMI was created so that we have a cross-cultural measure of socioeconomic status. In this analysis, our main exposure variable is stunting and WAMI is included in order to adjust for differing socioeconomic status across children and sites.*

The HOME inventory needs more explanation

***Response:*** *Thank you so much for your comment. We have added more about the HOME inventory in the methods section.*

The transferrin receptor is said to be important for growth so may also be a cause of stunting. Since they have previously reported the transferrin receptor as a risk factor for cognitive development it should not be one of the results mentioned in the abstract.

***Response****: In the MAL-ED study, transferrin receptor has been found to relate to both cognitive development and growth. Growth and cognitive development share many contributing factors; therefore, it can be challenging to tease out causal factors. This is a recognized challenge to observational studies, and we have controlled for these factors to the greatest extent possible. We have now revised the results of the abstract and the sentence on transferrin receptor has been deleted from the abstract.*

For the analysis of risk factors for early stunting it is not clear what the outcome is. Is it first measure of stunting within the first 2 years? Does it make sense to analyse this whole time period together? Risk factors for stunting in the first few months are likely to be different from later onset.

***Response****: Thank you for the question. Yes, the outcome of the risk factors for stunting analysis was first identification of stunting, and in this analysis, we utilized only those variables available at birth. We agree with you that some of the risk factors may vary based on the time of onset of stunting. However, most of the risk factors identified in the previous studies in the same settings remained consistent irrespective of time of onset of stunting in young children. Data from the MAL-ED study showed that maternal height and low birth weight contribute most in the development of stunting in children living in resource-poor settings [1].*

Figure 2 looks odd as it implies that no child was stunted at birth. Some left censoring is needed

***Response:*** *In this analysis we have only included those children who were not stunted at birth (i.e., at risk of ‘failure’ (stunting)).*

The analysis is based on 60% of the children enrolled. This loss to follow-up and any bias it may have introduced need to be discussed.

***Response:*** *Thank you for this statement. We compared the children who were included in the analysis and those who were excluded from the analysis, and found that the differences were rarely statistically significant at the site level. We have added a sentence about this to the limitations paragraph and please see the attached* *Supplemental Table 2.*

Other points:

Please avoid non-standard abbreviations in tables and figures

***Response:*** *Thank you so much. We have added non-standard abbreviations to the table foot note.*

Line 158: why multiply by 365?

***Response:*** *We have calculated the incidence of illness per year per child. That is why it was multiplied by 365.*

The conclusions call for a comparison with high income settings, but the causes of stunting in these settings are likely to be very different.

***Response:*** *We agree that the causes of stunting are likely to be very different in high income settings, and it would be interesting to explore the relationship between timing of stunting and cognitive development in higher income settings to determine if the relationships are similar or different.*

Reviewer #2: This manuscript presents an analysis of the MAL-ED LIMC multicountry cohort to explore the important association between early nutrition and later cognitive function. It largely confirms the previous findings that poor growth in early life is associated with poor cognitive development, also previously known information on timing and persistence of stunting. It controls for other contributing factors.

The writing is clear and precise and the data and findings well presented. The statistical methods used (though not my expertise) seem appropriate.

Although the findings are not particularly novel or unexpected, this is a useful addition to the literature. It strength is the multicountry approach, although given that the settings were so different, it might be helpful to explore whether there was interaction between site and any of the variables in the models and to stratify the analysis where appropriate.

***Response:*** *Thank you so much for your comments. We have run the model by site and combined the results in a figure. Please find the figure below:*

In the discussion the recommendation that the analysis could help with identification of specific higher risk groups for targeting misses some important principles (about absolute versus relative risk, distribution of the risk factor (50:50!) and equity) when suggesting eg that males could be targeted for interventions. Whilst males might be at higher risk in this analysis, an intervention which excludes female infants would exclude a very high proportion of infants who would develop stunting, be inequitable, and in any case, many of the interventions might be applied before the sex of the infant is known.

***Response:*** *We have edited the discussion to reflect the reviewer’s excellent points.*

 Reviewer #3: This paper analyzed the prospective birth cohort data from 6 LMICs – MAL-ED cohort study, to study the association between early-onset persistent stunting in children and their cognitive development at 5 years of age. In more detail, they grouped children into five categories based on their growth path: 1) never stunted, 2) early-onset persistent stunted if stunted prior to 6 months old, 3) early-onset recovered if stunted prior to 6 months old but not stunted at 60 months; 4) late-onset persistent (first stunted at 7-24 months and persisting at 60 months), 5) late onset recovered (first stunted at 7-24 months and not stunted at 60 months).  They ran linear regressions with different controls between these stunting categories and children’s development measured by the standardized Wechsler Preschool Primary Scales of Intelligence (WPPSI). They found, after controlling a set of social-economic characteristics, environmental variables, early-onset persistently stunted children had significantly lower cognitive scores by 2.1 than those who were never stunted. In addition, they found micronutrient plasma transferrin receptor (TfR) was also negatively associated with cognitive development.  The authors also identified the factors associated with early-onset stunting, and found males, lower birth weight, and untreated drinking water and family income and maternal height all contributed to early onset of stunting.
I think the paper is well written, the data is especially valuable and unique in the sense of similar design in different LMICs and incorporation of rich information on demographics and exposures of children over time, confounding social-economic background and environment factors. Finding on the adverse associations of early-onset stunting (before 6 months) has important policy implications. However, I do have following comments that hopefully can help authors to improve their manuscript:
1.  These six countries are very different in terms of stunting rate, children’s cognitive development, and socio-economic development. I wonder if these associations mainly come from cross-country variation, or within-country variation? I wish I could see analysis of heterogeneity in these associations across study sites. Will this same association hold within each country?

***Response:*** *Thank you so much for your comments. We have run the model by site and combined the results in a figure. Please find the figure below:*

2. Sample attrition is always an issue in prospective birth cohort studies. From Figure 1 we see a big attrition after dropping those without height data at 24 or 60 months. I wish authors could provide a table to compare the basic statistics between the final sample and the original sample, to give readers an idea of sample attrition, and acknowledge that in the discussion.

***Response:*** *Thank you for this question. We compared the children who were included in the analysis and those who were excluded from the analysis, and found that the differences were rarely statistically significant at the site level. We have added a sentence about this to the limitations paragraph and please see the attached* *Supplemental Table 2.*

3. Finding early-onset persistent stunting alone is significantly associated with lower WPPSI scores is interesting. This seems to imply children who recover from stunting after 6 months would do no worse in cognitive development at 5 year old. I wonder if authors could provide more analysis on catching-up, for example what factors contribute to catch up and the implications of policy aid.

***Response:*** *Thank you very much for this insightful comment. We completely agree with you in this point, and have already been working on this topic. We are drafting another manuscript on factors contributing to catch up growth in children. Hopefully, we will be able to submit the manuscript early next year.*

Minor comments:
1. Please report R2 for each model in Table 2.

***Response:*** *Thank you for the feedback. We have included the R^2^ value for each model in the Table 2.*

6. PLOS authors have the option to publish the peer review history of their article ([what does this mean?](https://nam01.safelinks.protection.outlook.com/?url=https%3A%2F%2Fjournals.plos.org%2Fplosone%2Fs%2Feditorial-and-peer-review-process%23loc-peer-review-history&data=02%7C01%7Clem118%40psu.edu%7Cc4667a97aa5648cc0b1008d76848179d%7C7cf48d453ddb4389a9c1c115526eb52e%7C0%7C1%7C637092531192192619&sdata=7iqyqdoVDBdRGqW2qacjWKgpmLCZdZ04LWXcyVHRc7A%3D&reserved=0)). If published, this will include your full peer review and any attached files.

If you choose “no”, your identity will remain anonymous but your review may still be made public.

**Do you want your identity to be public for this peer review?** For information about this choice, including consent withdrawal, please see our [Privacy Policy](https://nam01.safelinks.protection.outlook.com/?url=https%3A%2F%2Fwww.plos.org%2Fprivacy-policy&data=02%7C01%7Clem118%40psu.edu%7Cc4667a97aa5648cc0b1008d76848179d%7C7cf48d453ddb4389a9c1c115526eb52e%7C0%7C1%7C637092531192202609&sdata=LmrHBNYdhlNN9CygBaSkT%2F%2BL0JODSkPXcLqtb6Lmf2s%3D&reserved=0).

Reviewer #1: No

Reviewer #2: No

1.	Investigators M-EN. Childhood stunting in relation to the pre-and postnatal environment during the first 2 years of life: The MAL-ED longitudinal birth cohort study. PLoS medicine. 2017;14(10):e1002408.

2.	Yori PP, Lee G, Olórtegui MP, Chávez CB, Flores JT, Vasquez AO, et al. Santa Clara de Nanay: the MAL-ED cohort in Peru. Clinical Infectious Diseases. 2014;59(suppl_4):S310-S6.

3.	Turab A, Soofi SB, Ahmed I, Bhatti Z, Zaidi AK, Bhutta ZA. Demographic, socioeconomic, and health characteristics of the MAL-ED network study site in rural Pakistan. Clinical Infectious Diseases. 2014;59(suppl_4):S304-S9.

4.	Shrestha PS, Shrestha SK, Bodhidatta L, Strand T, Shrestha B, Shrestha R, et al. Bhaktapur, Nepal: the MAL-ED birth cohort study in Nepal. Clinical Infectious Diseases. 2014;59(suppl_4):S300-S3.

5.	Mduma ER, Gratz J, Patil C, Matson K, Dakay M, Liu S, et al. The etiology, risk factors, and interactions of enteric infections and malnutrition and the consequences for child health and development study (MAL-ED): description of the Tanzanian site. Clinical Infectious Diseases. 2014;59(suppl_4):S325-S30.

6.	Lima AA, Oriá RB, Soares AM, Filho JQ, de Sousa Jr F, Abreu CB, et al. Geography, population, demography, socioeconomic, anthropometry, and environmental status in the MAL-ED cohort and case-control study sites in Fortaleza, Ceará, Brazil. Clinical Infectious Diseases. 2014;59(suppl_4):S287-S94.

7.	John SM, Thomas RJ, Kaki S, Sharma SL, Ramanujam K, Raghava MV, et al. Establishment of the MAL-ED birth cohort study site in Vellore, southern India. Clinical Infectious Diseases. 2014;59(suppl_4):S295-S9.

8.	Bessong PO, Nyathi E, Mahopo TC, Netshandama V. Development of the Dzimauli community in Vhembe District, Limpopo province of South Africa, for the MAL-ED cohort study. Clinical Infectious Diseases. 2014;59(suppl_4):S317-S24.

9.	Ahmed T, Mahfuz M, Islam MM, Mondal D, Hossain MI, Ahmed AS, et al. The MAL-ED cohort study in Mirpur, Bangladesh. Clinical Infectious Diseases. 2014;59(suppl_4):S280-S6.

10.	Ruan-Iu L, Pendergast LL, Rasheed M, Tofail F, Svensen E, Maphula A, et al. Assessing Early Childhood Fluid Reasoning in Low-and Middle-Income Nations: Validity of the Wechsler Preschool and Primary Scale of Intelligence Across Seven MAL-ED Sites. Journal of Psychoeducational Assessment. 2019:0734282919850040.
